# Out of Hours Work: A Trainee-Led Review

**DOI:** 10.1192/bjo.2024.477

**Published:** 2024-08-01

**Authors:** Charlotte Hall, Drew Garnham-McEwan

**Affiliations:** 1 Sheffield Health & Social Care, Sheffield, United Kingdom; 2 Rotherham Doncaster and South Humber NHS Foundation Trust, Rotherham, United Kingdom

## Abstract

**Aims:**

The 2016 Junior Doctor’s contract offers guidance as to the rest periods needed during non-resident on-calls (NROCs). The Rotherham, Doncaster and South Humber (RDaSH) NHS Foundation Trust currently works on a NROC trainee rota. NROC work undertaken is monitored via a log form, returned by the trainee after their shift. A retrospective audit was completed with only a 28% return rate of log forms. Though anecdotal evidence suggested inadequate rest and high workloads during on-calls, due to low engagement in monitoring formal data was lacking. Therefore, a trainee-led prospective audit was designed to formally monitor on-call workload over a period of 4 weeks.

The main aim of this project was to review the average amount of hours worked during an NROC shift and compare achieved rest periods against agreed standards (derived from 2016 contract). These standards indicate that 90% of shifts should achieve 8 hours rest in 24 hours and 5 hours continuous rest between 22:00–07:00. In order to accomplish this we first aimed to increase the return of completed on-call log forms to 75%.

**Methods:**

Work was predominantly concentrated around increasing return rate of the log forms including: running teaching sessions, regional promotion, and sending daily reminder emails to return the forms. These forms were then reviewed and analysed.

**Results:**

Across the 4 week audit period, the return rate of log forms was 95%, compared with the previous return rate of 28%. Average hours worked across all three localities exceeded the expected hours by RDaSH. When compared with the standards outlined, 1 in 3 shifts in Rotherham, 1 in 5 in Doncaster and 1 in 4 in South Humber did not achieve contractual rest periods. Out of these, not reaching 5 hours continuous rest was the most common reason for not meeting contractual rest periods.

**Conclusion:**

RDaSH worked collaboratively with trainees to generate a number of interventions to mitigate the breaches in rest periods including: creation of a new clinical role to filter calls, reviewing the suitability of the NROC rota and increasing pay to reflect the increased workload. There is currently work underway to redesign the rota.

This audit highlights the importance of prioritising regular reviews of NROC work to ensure the safety of both staff and patients through achieving adequate rest periods.

